# Salbutamol use in relation to maintenance bronchodilator efficacy in COPD: a prospective subgroup analysis of the EMAX trial

**DOI:** 10.1186/s12931-020-01451-8

**Published:** 2020-10-22

**Authors:** F. Maltais, I. P. Naya, C. F. Vogelmeier, I. H. Boucot, P. W. Jones, L. Bjermer, L. Tombs, C. Compton, D. A. Lipson, E. M. Kerwin

**Affiliations:** 1 Centre de Pneumologie, Institut Universitaire de Cardiologie et de Pneumologie de Québec, Université Laval, Québec, QC Canada; 2 GSK, Brentford, Middlesex, UK; 3 RAMAX Ltd, Bramhall, Cheshire, UK; 4 Department of Medicine, Pulmonary and Critical Care Medicine, University Medical Center Giessen and Marburg, Philipps-Universität Marburg, Member of the German Center for Lung Research (DZL), Marburg, Germany; 5 Respiratory Medicine and Allergology, Lund University, Lund, Sweden; 6 Precise Approach Ltd, contingent worker on assignment at GSK, Stockley Park West, Uxbridge, Middlesex, UK; 7 Respiratory Clinical Sciences, GSK, Collegeville, PA USA; 8 Perelman School of Medicine, University of Pennsylvania, Philadelphia, PA USA; 9 Clinical Research Institute of Southern Oregon, Medford, OR USA

**Keywords:** (3–10): dual bronchodilators, COPD, Symptoms, Lung function, Rescue therapy, SABA, Salbutamol

## Abstract

**Background:**

Short-acting β_2_-agonist (SABA) bronchodilators help alleviate symptoms in chronic obstructive pulmonary disease (COPD) and may be a useful marker of symptom severity. This analysis investigated whether SABA use impacts treatment differences between maintenance dual- and mono-bronchodilators in patients with COPD.

**Methods:**

The Early MAXimisation of bronchodilation for improving COPD stability (EMAX) trial randomised symptomatic patients with low exacerbation risk not receiving inhaled corticosteroids 1:1:1 to once-daily umeclidinium/vilanterol 62.5/25 μg, once-daily umeclidinium 62.5 μg or twice-daily salmeterol 50 μg for 24 weeks. Pre-specified subgroup analyses stratified patients by median baseline SABA use (low, < 1.5 puffs/day; high, ≥1.5 puffs/day) to examine change from baseline in trough forced expiratory volume in 1 s (FEV_1_), change in symptoms (Transition Dyspnoea Index [TDI], Evaluating Respiratory Symptoms-COPD [E-RS]), daily SABA use and exacerbation risk. A post hoc analysis used fractional polynomial modelling with continuous transformations of baseline SABA use covariates.

**Results:**

At baseline, patients in the high SABA use subgroup (mean: 3.91 puffs/day, *n* = 1212) had more severe airflow limitation, were more symptomatic and had worse health status versus patients in the low SABA use subgroup (0.39 puffs/day, *n* = 1206). Patients treated with umeclidinium/vilanterol versus umeclidinium demonstrated statistically significant improvements in trough FEV_1_ at Week 24 in both SABA subgroups (59–74 mL; *p* < 0.001); however, only low SABA users demonstrated significant improvements in TDI (high: 0.27 [*p* = 0.241]; low: 0.49 [*p* = 0.025]) and E-RS (high: 0.48 [*p* = 0.138]; low: 0.60 [*p* = 0.034]) scores. By contrast, significant reductions in mean SABA puffs/day with umeclidinium/vilanterol versus umeclidinium were observed only in high SABA users (high: − 0.56 [*p* < 0.001]; low: − 0.10 [*p* = 0.132]). Similar findings were observed when comparing umeclidinium/vilanterol and salmeterol. Fractional polynomial modelling showed baseline SABA use ≥4 puffs/day resulted in smaller incremental symptom improvements with umeclidinium/vilanterol versus umeclidinium compared with baseline SABA use < 4 puffs/day.

**Conclusions:**

In high SABA users, there may be a smaller difference in treatment response between dual- and mono-bronchodilator therapy; the reasons for this require further investigation. SABA use may be a confounding factor in bronchodilator trials and in high SABA users; changes in SABA use may be considered a robust symptom outcome.

**Funding:**

GlaxoSmithKline (study number 201749 [NCT03034915]).

## Introduction

Some patients with chronic obstructive pulmonary disease (COPD) using long-acting muscarinic antagonists (LAMAs) or long-acting β_2_-agonists (LABAs) also use frequent short-acting β_2_-agonist (SABA) rescue therapy [1]. An analysis of more than 23,000 patients from 23 clinical trials of mono- and dual- bronchodilators demonstrated that patients across a variety of COPD severities used approximately 4 SABA puffs/day [2]. Patients may use high levels of SABA for a number of reasons, including having poorly controlled COPD due to suboptimal prescribing of maintenance therapy, having a mild exacerbation, poor adherence or responsiveness to maintenance therapy, or a lack of access to appropriate therapy. SABA use tends to increase with increasing COPD severity; in patients who were receiving a single LAMA or LABA bronchodilator in routine US clinical practice, a mean SABA use of 3.3 puffs/day was reported in patients with less severe airflow limitation (≥50% predicted forced expiratory volume in 1 s [FEV_1_]), compared with 5.2 puffs/day in patients with more severe airflow limitation (< 50% predicted FEV_1_) [3]. This suggests that patients with COPD who frequently use SABA may be inadequately treated with their current maintenance therapies [4].

High supplementary SABA use is a marker of an increased risk of exacerbations and hospitalisation and is associated with significant economic costs [5, 6]. In COPD clinical trials, reductions in daily SABA use (puffs/day) have also been shown to positively correlate with mean improvements in lung function, exacerbation rates and health-related quality of life [7]. Therefore, the assessment of rescue medication use in patients with COPD is likely to be a useful measure of changes in symptom burden in clinical trials and routine clinical practice [7].

Although rescue medication use may be a useful indication of symptom severity there is also some evidence to suggest that high levels of rescue medication use may affect the assessment of patient-perceived symptom severity. A previous post hoc analysis of two large, 6-month bronchodilator trials suggested that symptomatic patients with high SABA use (≥3.6 puffs/day) perceive a lower benefit of treatment differences between maintenance bronchodilator therapy and placebo on dyspnoea (measured using the transition dyspnoea index [TDI]) compared with low SABA users (< 3.6 puffs/day) [1]. This finding may have important implications for the design of clinical trials; therefore, this prespecified analysis was performed to investigate the potential confounding effect of SABA use on treatment differences observed between a LAMA/LABA combination and LAMA or LABA monotherapy in more detail.

The Early MAXimisation of bronchodilation for improving COPD stability (EMAX) trial examined the benefits of dual bronchodilation with the LAMA/LABA combination of umeclidinium/vilanterol (UMEC/VI) versus the LAMA UMEC and the LABA salmeterol (SAL) in symptomatic patients at low exacerbation risk who were not receiving inhaled corticosteroids (ICS) [8]. The primary analysis demonstrated consistent incremental benefits of UMEC/VI compared with both monotherapies on lung function and symptoms [8]. The objective of this pre-specified analysis of the EMAX trial was to prospectively investigate whether differences in SABA use at baseline are associated with differences in lung function and symptomatic treatment responses to dual- versus mono-bronchodilation in symptomatic patients with COPD. As such, subgroup analyses of high and low SABA users were conducted to compare treatment differences in lung function and symptoms for each subgroup.

## Methods

### Study design and treatments

This was a pre-specified analysis of the multicentre, randomised, double-blind, double-dummy, 3-arm parallel group EMAX trial (NCT03034915; GSK study number 201749). Full details of the study methodology have been published previously [8]. Briefly, patients were randomised 1:1:1 to once-daily UMEC/VI 62.5/25 μg delivered via the ELLIPTA inhaler, once-daily UMEC 62.5 μg via the ELLIPTA inhaler, and twice-daily SAL 50 μg via the DISKUS inhaler, for 24 weeks.

### Patients

Patients were ≥ 40 years of age with a current diagnosis of COPD according to the American Thoracic Society/European Respiratory Society definition [9], were current or former smokers with ≥10 pack-years of smoking history, had a pre- and post-salbutamol FEV_1_/forced vital capacity ratio < 0.7 and post-salbutamol FEV_1_ of ≥30–≤80% of predicted (Global Initiative for COPD [GOLD] grade 2/3 lung function), a COPD Assessment Test (CAT) score ≥ 10, had ≤1 moderate exacerbation and no severe exacerbations in the prior year and were ICS free for ≥6 weeks and LAMA/LABA free for ≥2 weeks prior to 4-week run-in period. During the run-in period, patients were limited to a maximum of one bronchodilator maintenance therapy with either a LAMA or LABA. As-needed salbutamol was permitted throughout all study phases but was not permitted within the 4 h prior to spirometry testing.

### Endpoints and assessments

Endpoints assessed in this pre-specified analysis included change from baseline in FEV_1_ at Week 24, self-administered computerised-TDI (SAC-TDI) at Week 24, change from baseline in Evaluating Respiratory Symptoms-COPD (E-RS) at Weeks 21–24, and global assessment of disease severity (GADS; change from baseline rated on a seven-point Likert scale of ‘much better’, ‘better’, ‘slightly better’, ‘no change’, ‘slightly worse’, ‘worse’, ‘much worse’) at Week 24. Daily SABA (salbutamol) use (puffs/day) was reported across the 24 weeks.

Analysis of trough FEV_1_ responders, defined as patients with an improvement from baseline of ≥100 mL, was performed post hoc. SAC-TDI and E-RS responders were analysed prospectively with responders defined as patients with a ≥ 1-unit and ≥ 2-point improvement from baseline, respectively [10, 11]. Risk of a first moderate or severe exacerbation up to Day 168 was also determined. Safety outcomes included incidence of adverse events (AEs) and serious AEs (SAEs).

To use more of the information available in the range of SABA use, fractional polynomial modelling was performed as a post hoc analysis. Changes in FEV_1_ and SAC-TDI at Week 24, SABA use (puffs/day) at Weeks 1–24, and E-RS at Weeks 21–24, were assessed using fractional polynomial modelling with continuous transformations of baseline SABA use as a covariate to understand if and at what level of baseline SABA use the efficacy differences between the three maintenance bronchodilator regimens were impacted.

### Statistical analysis

Subgroup analyses in high and low SABA users were performed prospectively with the intent-to-treat (ITT) population of the EMAX trial stratified by median baseline SABA use (low, < 1.5 puffs/day; high, ≥1.5 puffs/day). Comparisons between baseline characteristics of low and high SABA subgroups were descriptive only.

Trough FEV_1_ and patient-reported outcomes were analysed using mixed model repeated measures analyses adjusted for covariates of baseline score, geographical region, number of bronchodilators per day during run-in, visit/4-weekly period, treatment, visit/4-weekly period by baseline and visit/4-weekly period by treatment interactions. Data are presented as least squares (LS) mean and LS mean change from baseline, with estimated treatment differences and 95% confidence intervals (CIs). Responder analyses with corresponding odds ratios (OR) and 95% CIs were performed using a generalised linear mixed model with covariates of baseline score, number of bronchodilators per day during run-in, geographical region, number of bronchodilators per day during run-in, visit/4-weekly period by baseline and visit/4-weekly period by treatment interactions. For the GADS, ordered ORs of a better response category (‘slightly better’, ‘better’ or ‘much better’) on the seven-point scale at each visit were analysed using a generalised linear model with covariates of treatment, geographical region and the number of bronchodilators per day during run-in. Time to study treatment withdrawal and first exacerbation hazard ratios (HR) and 95% CIs were based on Cox proportional hazards model with covariates of treatment, number of bronchodilators per day during run-in, and geographical region.

For fractional polynomial modelling, transformation of baseline SABA use were assessed using a fitted mixed model repeated measures with additional covariates of baseline endpoint value, geographical region, number of bronchodilators per day during run-in, visit/4-weekly period, treatment, first-degree fractional polynomial (FP1), FP2, visit/4-weekly period by baseline, visit/4-weekly period by treatment, FP1-treatment and FP2-treatment interactions.

## Results

### Patient disposition and demographics

Of the 2425 patients in the ITT population, 1212 patients were in the high baseline SABA (≥1.5 puffs/day) subgroup (UMEC/VI: 415, UMEC: 401, SAL: 396), and 1206 patients were in the low baseline SABA (< 1.5 puffs/day) subgroup (UMEC/VI: 395, UMEC: 399, SAL: 412). Seven patients had no available SABA use data and were excluded from this analysis. Mean baseline SABA use was ten times higher in the high SABA use subgroup (mean 3.91 puffs/day) compared with the low SABA subgroup (mean 0.39 puffs/day). Approximately one-third of the ITT population and the majority of patients in the low SABA use subgroup were not using any SABA at baseline whereas 18% of the ITT were using ≥4 puffs/day (Supplementary Fig. 1). More patients in the high SABA use subgroup were current smokers, were not receiving maintenance treatment during run-in, and had more severe airflow limitation and a worse symptom burden and health status at baseline compared with the low SABA use subgroup (Table 1). However, within each of the prespecified SABA use subgroups, the treatment groups were generally well-balanced in terms of age, sex, smoking pack years, airflow limitation, symptom burden and health status impact at baseline (Table 1). In the high SABA use subgroup, the proportion of patients who withdrew from study treatment was 18–22% on monotherapies compared with 12% on UMEC/VI; consequently the HR for treatment withdrawal was significantly lower with UMEC/VI versus either monotherapy. In contrast, for the low SABA use subgroup, treatment withdrawal rates were 12–15% on all regimens and there was no significant difference between treatments in the risk of study treatment withdrawal (Supplementary Table 1).

**Table 1:** Patient demographics and baseline characteristics by baseline SABA use

Characteristic	High baseline SABA (≥1.5 puffs/day)				Low baseline SABA (< 1.5 puffs/day)			
	UMEC/VI (***n*** = 415)	UMEC (***n*** = 401)	SAL (***n*** = 396)	Total (***N*** = 1212)	UMEC/VI (***n*** = 395)	UMEC (***n*** = 399)	SAL (***n*** = 412)	Total (***N*** = 1206)
Age, years, mean (SD)	63.8 (8.6)	63.8 (8.4)	63.1 (8.5)	63.5 (8.5)	65.6 (8.1)	66.0 (8.4)	65.6 (8.4)	65.7 (8.3)
Female, n (%)	168 (40)	179 (45)	182 (46)	529 (44)	149 (38)	146 (37)	159 (39)	454 (38)
Current smoker at screening, n (%)	205 (49)	222 (55)	233 (59)	660 (54)	187 (47)	171 (43)	180 (44)	538 (45)
Smoking pack-years, mean (SD)	49.6 (28.1)	48.1 (26.8)	46.8 (24.7)	48.2 (26.6)	49.3 (27.4)	47.4 (25.1)	49.4 (26.8)	48.7 (26.4)
Moderate COPD exacerbation history in prior year^a^, n (%)	63 (15)	59 (15)	66 (17)	188 (16)	60 (15)	63 (16)	79 (19)	202 (17)
Duration of COPD, years, mean (SD)	9.0 (6.6)	8.1 (5.9)	9.0 (6.2)	8.7 (6.3)	8.5 (7.2)	7.6 (6.0)	7.6 (7.1)	7.9 (6.8)
No maintenance treatment during run-in, %	147 (35)	144 (36)	148 (37)	439 (36)	103 (26)	105 (26)	101 (25)	309 (26)
Post-salbutamol % predicted FEV_1_, mean (SD)	53.1 (13.1)	53.2 (12.6)	53.0 (12.9)	53.1 (12.8)	56.8 (12.2)	58.6 (12.2)	58.1 (12.2)	57.8 (12.2)
GOLD spirometric grade^b^, n (%)								
2	240 (58)	233 (58)	221 (56)	694 (57)	277 (70)	294 (74)	300 (73)	871 (72)
3	175 (42)	166 (42)	175 (44)	516 (43)	118 (30)	103 (26)	111 (27)	332 (28)
BDI score, mean (SD)	6.8 (1.9)	6.7 (2.0)	6.8 (1.9)	6.7 (1.9)	7.2 (1.8)	7.3 (1.8)	7.3 (1.7)	7.3 (1.8)
Baseline E-RS total score, mean (SD)	12.4 (5.5)	12.7 (5.8)	12.4 (5.6)	12.5 (5.6)	8.9 (5.1)	8.8 (5.2)	8.5 (5.2)	8.7 (5.2)
Baseline rescue salbutamol, puffs/day, mean (SD)	3.90 (2.5)	3.87 (2.2)	3.98 (2.4)	3.91 (2.39)	0.37 (0.4)	0.37 (0.4)	0.43 (0.5)	0.39 (0.45)
Baseline CAT score, mean (SD)	20.8 (5.9)	20.7 (6.3)	21.0 (6.7)	20.8 (6.3)	17.3 (5.4)	17.8 (5.7)	17.6 (5.5)	17.6 (5.5)
Baseline SGRQ total score, mean (SD)	48.9 (15.8)	49.1 (15.7)	49.9 (16.3)	49.3 (15.9)	39.8 (15.1)	40.9 (15.4)	39.5 (14.6)	40.1 (15.0)
Any vascular comorbidity, n (%)	233 (56)	217 (54)	218 (55)	668 (55)	209 (53)	215 (54)	230 (56)	654 (54)
Any cardiac comorbidity, n (%)	60 (14)	71 (18)	58 (15)	189 (16)	50 (13)	64 (16)	59 (14)	173 (14)
Coronary artery disease	47 (11)	53 (13)	35 (9)	135 (11)	33 (8)	46 (12)	45 (11)	124 (10)
Arrhythmia	17 (4)	23 (6)	19 (5)	59 (5)	18 (5)	21 (5)	17 (4)	56 (5)
Congestive heart failure	6 (1)	8 (2)	11 (3)	25 (2)	6 (2)	9 (2)	8 (2)	23 (2)

^a^Number of exacerbations requiring oral or systemic corticosteroids and/or antibiotics (moderate) in 12 months prior to screening (patients with > 1 moderate exacerbation or with a severe exacerbation [requiring hospitalisation] were excluded); ^b^an additional 4 (< 1%) patients with GOLD grade 1 were randomised (UMEC *n =* 3; SAL *n =* 1)

*BDI* Baseline Dyspnoea Index, *CAT* COPD Assessment Test, *COPD* chronic obstructive pulmonary disease, *E-RS* Evaluating Respiratory Symptoms: *COPD, FEV*_*1*_ forced expiratory volume in 1 s, *GOLD* Global Initiative for Chronic Obstructive Lung Disease, *ITT* intent-to-treat, *SD* standard deviation, *SABA* short-acting β_2_-agonist, *SAL* salmeterol, *SGRQ* St George’s Respiratory Questionnaire, *UMEC* umeclidinium, *VI* vilanterol

### Lung function

**Fig. 1:**
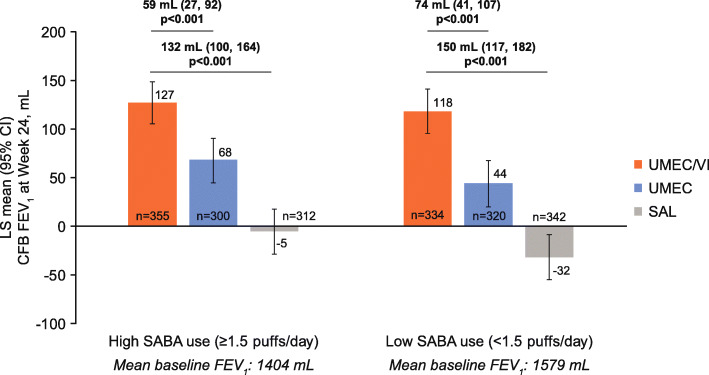
Change from baseline in trough FEV_1_ at Week 24 by baseline SABA use^a.^ *CFB* change from baseline, *CI* confidence interval, *FEV*_*1*_ forced expiratory volume in 1 s, *LS* least squares, *SABA* short-acting β_2_-agonist, *SAL* salmeterol, *UMEC* umeclidinium, *VI* vilanterol. ^a^Mean SABA use was 3.91 puffs/day and 0.39 puffs/day in the high and low SABA use subgroups, respectively

Patients treated with UMEC/VI demonstrated significant improvements from baseline in trough FEV_1_ versus UMEC and SAL in both the high and low SABA use subgroups (Fig. 1). The magnitude of trough FEV_1_ improvement with UMEC/VI versus UMEC and SAL was similar in the high and low SABA use subgroups (UMEC/VI vs UMEC: 59 and 74 mL; UMEC/VI vs SAL: 132 and 150 mL, respectively; all *p* < 0.001, Fig. 1) and there was no evidence of a significant interaction between baseline SABA use (< 1.5 or ≥ 1.5 puffs/day) and treatment difference on trough FEV_1_ (*p* = 0.986). Patients receiving UMEC/VI were significantly more likely to be a trough FEV_1_ responder after 24 weeks than those receiving UMEC or SAL in both the high and low SABA use subgroups (Table 2).

**Table 2:** Proportion of trough FEV_1_ SAC-TDI, E-RS responders and GADS assessment

	High baseline SABA (≥1.5 puffs/day)			Low baseline SABA (< 1.5 puffs/day)		
	UMEC/VI	UMEC	SAL	UMEC/VI	UMEC	SAL
Trough FEV_1_ responders^a^, n/N (%)	193/411 (47)	131/391 (34)	90/387 (23)	173/383 (45)	114/384 (30)	80/398 (20)
UMEC/VI vs comparator odds ratio (95% CI)	–	1.81 (1.35, 2.42); *p <* 0.001	3.07 (2.25, 4.21); *p <* 0.001	–	2.03 (1.49, 2.76); *p <* 0.001	3.44 (2.48, 4.77); *p <* 0.001
SAC-TDI responders^b^, n/N (%)	220/415 (53)	170/398 (43)	164/394 (42)	183/389 (47)	162/398 (41)	166/412 (40)
UMEC/VI vs comparator odds ratio (95% CI)	–	1.52 (1.14, 2.02); *p* = 0.004	1.60 (1.20, 2.13); *p* = 0.001	–	1.34 (1.01, 1.79); *p* = 0.044	1.37 (1.03, 1.82); *p* = 0.031
E-RS responders^c^, n/N (%)	163/415 (39)	124/401 (31)	122/396 (31)	127/394 (32)	95/399 (24)	95/412 (23)
UMEC/VI vs comparator odds ratio (95% CI)	–	1.47 (1.10, 1.98); *p* = 0.010	1.47 (1.10, 1.98); *p* = 0.011	–	1.57 (1.14, 2.17); *p* = 0.006	1.59 (1.16, 2.19); *p* = 0.004
GADS^d^, n/N (%)	246/363 (68)	187/305 (61)	202/322 (63)	227/342 (66)	205/332 (62)	211/352 (60)
UMEC/VI vs comparator odds ratio (95% CI)	–	1.38 (1.05, 1.82); *p* = 0.020	1.26 (0.96, 1.65); *p* = 0.097	–	1.41 (1.08, 1.86); *p* = 0.013	1.55 (1.18, 2.04); *p* = 0.002

^a^Trough FEV_1_ responders were defined as patients with ≥100 mL trough FEV_1_ improvement from baseline at Week 24; ^b^SAC-TDI responders were defined as patients with a score ≥ 1 at Week 24; ^c^E-RS responders were defined as patients with ≥2-point improvement from baseline at Weeks 21─24; ^d^patients with a GADS response category of ‘slightly better’, ‘better’ or ‘much better’ at Week 24

*CI* confidence interval, *E-RS* Evaluating Respiratory Symptoms: *COPD, FEV*_*1*_ forced expiratory volume in 1 s, *GADS* global assessment of disease severity, *SABA* short-acting β_2_-agonist, *SAC-TDI* self-administered computerised Transition Dyspnoea Index, *SAL* salmeterol, *UMEC* umeclidinium, *VI* vilanterol

### Symptom outcomes

**Fig. 2:**
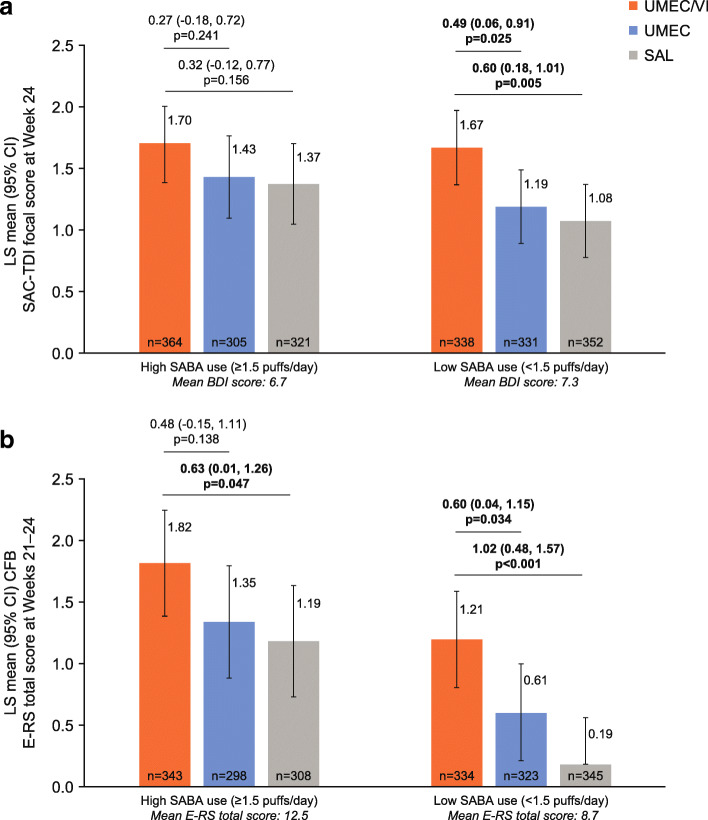
Mean (**a**) SAC-TDI (**b**) change from baseline E-RS total score by baseline SABA use^a^. *CFB *change from baseline, *CI *confidence interval, *ERS* Evaluating Respiratory Symptoms: COPD, *LS* least squares, *SABA* short-acting β_2_-agonist, *SAC-TDI* self-administered computerised Transition Dyspnoea Index, *SAL* salmeterol, *UMEC* umeclidinium, *VI* vilanterol. ^a^Mean SABA use was 3.91 puffs/day and 0.39 puffs/day in the high and low SABA use subgroups, respectively

In the high SABA use subgroup, numerical (but not statistically significant) improvements in SAC-TDI focal score were observed with UMEC/VI versus UMEC and SAL at Week 24 (Fig. 2a). In the low SABA use subgroup, the greater improvements in SAC-TDI for UMEC/VI versus either monotherapy reached significance (Fig. 2a). However, the odds of being a SAC-TDI responder at Week 24 were significantly greater with UMEC/VI versus UMEC and SAL in both the high and low SABA use subgroups (OR ranged from 1.34 to 1.60; *p* < 0.05, Table 2).

UMEC/VI provided numerically greater improvements in daily E-RS total score at Weeks 21–24 versus UMEC in both SABA use subgroups, although statistical significance was only reached in the low SABA use subgroup (Fig. 2b). Significant differences in E-RS total score were observed with UMEC/VI versus SAL for both high and low SABA use subgroups (Fig. 2b). The odds of being an E-RS responder at Weeks 21–24 were significantly greater with UMEC/VI versus both UMEC and SAL in both high and low SABA use subgroups (OR ranged from 1.47 to 1.59, *p* < 0.05, Table 2).

For the GADS, the majority of patients reported feeling ‘much better’, ‘better’ or ‘slightly better’ at Week 24 compared with baseline for all treatments in the high and low SABA use subgroups. The odds of a patient reporting a better response category were significantly greater with UMEC/VI versus UMEC for both high and low SABA use subgroups, and versus SAL for the low, but not the high SABA use subgroup (Table 2).

### SABA use

**Fig. 3:**
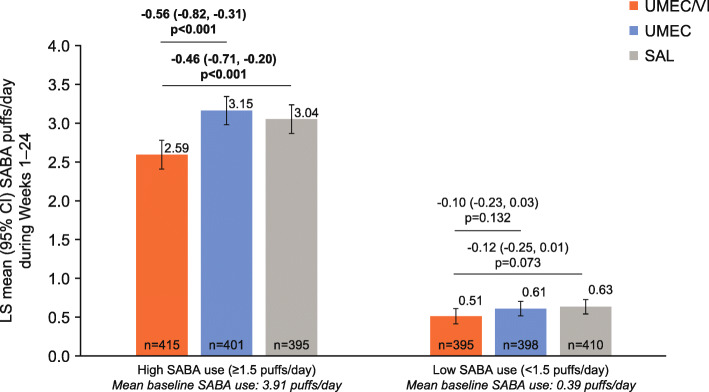
Mean SABA puffs/day across Weeks 1–24. *CI* confidence interval, *LS* least squares, *SABA* short-acting β_2_-agonist, *SAL* salmeterol, *UMEC* umeclidinium, *VI* vilanterol

Patients in the high baseline SABA use subgroup had higher on-treatment mean SABA use across Weeks 1–24 compared with the low baseline SABA use subgroup (Fig. 3). When comparing treatment benefits on SABA use, a significant reduction in the number of SABA puffs/day over 24 weeks was observed in the high SABA use subgroup receiving UMEC/VI versus UMEC and SAL (− 0.46 and − 0.56 puffs/day, respectively; both *p* < 0.001, Fig. 3). By contrast, in the low SABA use subgroup, patients receiving UMEC/VI showed no significant reduction in the number of SABA puffs/day over 24 weeks of treatment versus either monotherapy.

### Risk of a first exacerbation

**Fig. 4:**
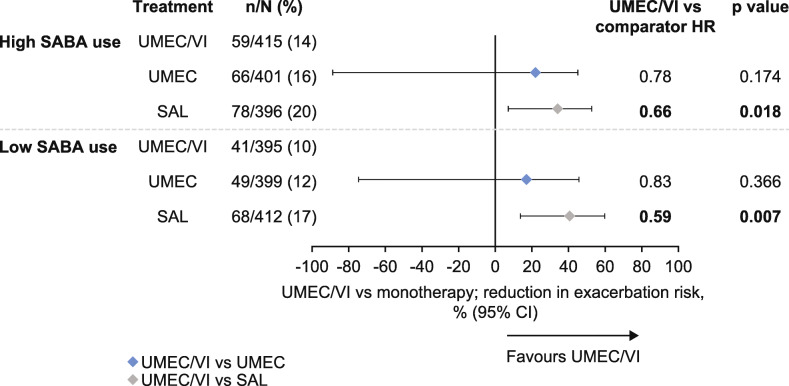
Risk of a first moderate/severe exacerbation up to Day 168. *CI* confidence interval, *HR* hazard ratio, *LS* least squares, *SABA* short-acting β_2_-agonist, *SAL* salmeterol, *UMEC* umeclidinium, *VI* vilanterol

For all treatment groups, the incidence of a first moderate or severe exacerbation event was numerically higher in the high SABA use subgroup than in the low SABA use subgroup for the duration of the study (Fig. 4). There was no significant difference in the risk of a moderate or severe exacerbation up to Day 168 with UMEC/VI versus UMEC in either subgroup (high SABA risk reduction [RR]: 22%, *p* = 0.174; low SABA RR: 17%, *p* = 0.366; Fig. 4). However, for UMEC/VI versus SAL there was a significantly lower risk of moderate or severe exacerbations in both subgroups (high SABA RR: 34%, *p* = 0.018; low SABA RR: 41%, *p* = 0.007).

### Sensitivity analysis by baseline SABA use

**Fig. 5:**
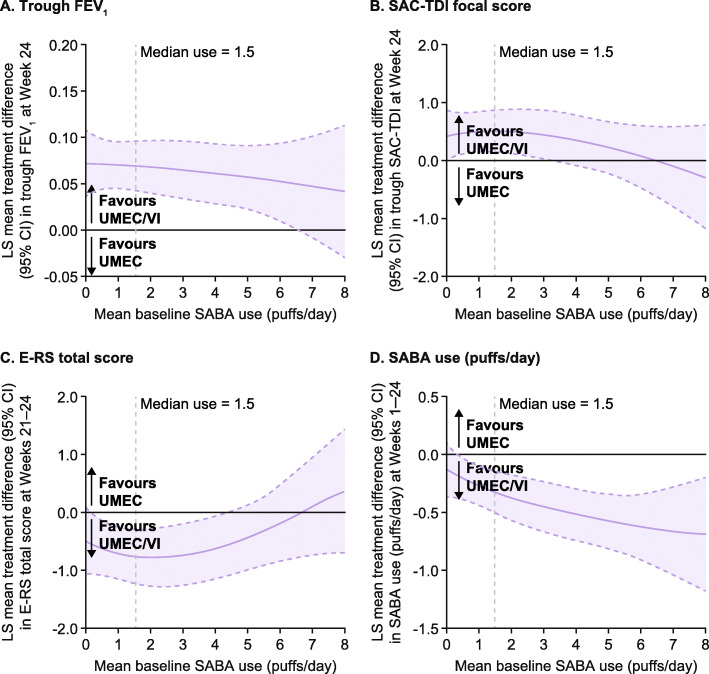
Relationship between baseline SABA use and treatment differences (UMEC/VI vs UMEC). *CI* confidence interval, *E-RS* Evaluating Respiratory Symptoms: COPD, *FEV1* forced expiratory volume in 1 s, *LS* least squares, *SABA* short-acting β_2_-agonist, *SAC-TDI *self-administered computerised Transition Dyspnoea Index, *UMEC* umeclidinium, *VI* vilanterol

**Fig. 6:**
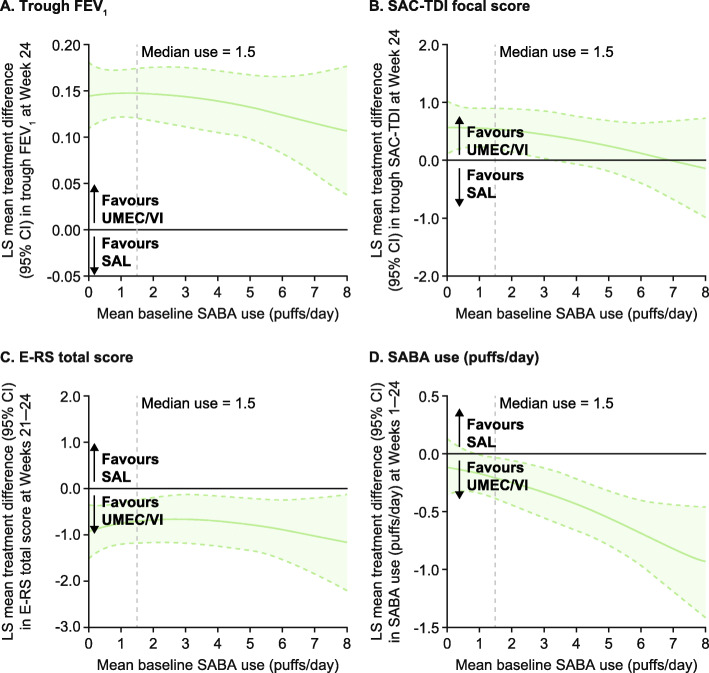
Relationship between baseline SABA use and treatment difference (UMEC/VI vs SAL). *CI* confidence interval, *E-RS* Evaluating Respiratory Symptoms: COPD, *FEV1* forced expiratory volume in 1 s, *LS* least squares, *SABA* short-acting β_2_-agonist, *SAC-TDI* self-administered computerised Transition Dyspnoea Index, *SAL* salmeterol, *UMEC* umeclidinium, *VI* vilanterol

A post hoc sensitivity analysis for the ITT population, using fractional polynomial modelling, suggested there were improvements from baseline in trough FEV_1_ at Week 24 in patients treated with UMEC/VI versus UMEC who were using less than approximately 6.5 puffs/day SABA at baseline (as the confidence intervals exclude the possibility of a zero treatment difference below 6.5 puffs/day) (Fig. 5a). Fractional polynomial modelling of UMEC/VI versus SAL suggested improvements from baseline in trough FEV_1_ at Week 24 regardless of baseline SABA use (Fig. 6a). The greatest improvements in SAC-TDI focal score at Week 24 and E-RS score at Weeks 21–24 for UMEC/VI versus UMEC were observed in patients with < 4 puffs/day SABA (Fig. 5b and c). When SABA use was greater than approximately 4 puffs/day, no additional benefits in SAC-TDI focal score or E-RS total score were noted for UMEC/VI over UMEC based on the confidence intervals including the possibility of a zero treatment difference (Fig. 5b and c). Similar findings were seen with UMEC/VI versus SAL for SAC-TDI focal score (Fig. 6b), whereas perceived symptom benefits between UMEC/VI and SAL for E-RS total score were generally similar across all levels of SABA use (Fig. 6c). The magnitude of treatment benefit on SABA use (puffs/day) for UMEC/VI versus both monotherapies increased with increasing baseline SABA use (Fig. 5d and 6d).

### Safety

The incidence of AEs, drug-related AEs, common AEs and SAEs was similar in all treatment groups in both the high and low SABA subgroups (Table 3). There were no treatment-related fatal AEs.

**Table 3:** Adverse events

	High baseline SABA (≥1.5 puffs/day)			Low baseline SABA (< 1.5 puffs/day)		
	UMEC/VI	UMEC	SAL	UMEC/VI	UMEC	SAL
**AE, n (%)**						
AE	157 (38)	170 (42)	156 (39)	158 (40)	146 (37)	158 (38)
Drug-related AE	9 (2)	16 (4)	11 (3)	20 (5)	21 (5)	16 (4)
AE leading to study withdrawal	12 (3)	21 (5)	14 (4)	20 (5)	15 (4)	12 (3)
**SAE, n (%)**						
Non-fatal SAE	25 (6)	17 (4)	22 (6)	21 (5)	14 (4)	16 (4)
Drug-related non-fatal SAE	0	0	0	0	0	0
Fatal SAE	1 (< 1)	2 (< 1)	0	3 (< 1)	2 (< 1)	0
Drug-related fatal SAE	0	0	0	0	0	0
**Most frequent AEs,**^**a**^ **n (%)**						
Nasopharyngitis	30 (7)	40 (10)	32 (8)	38 (10)	47 (12)	52 (13)
URTI	9 (2)	9 (2)	12 (3)	10 (3)	3 (< 1)	8 (2)
Influenza	12 (3)	5 (1)	9 (2)	8 (2)	4 (1)	9 (2)
Back pain	8 (2)	8 (2)	10 (3)	2 (< 1)	5 (1)	5 (1)
Cough	3 (< 1)	3 (< 1)	5 (1)	11 (3)	8 (2)	5 (1)
Bronchitis	8 (2)	7 (2)	8 (2)	3 (< 1)	4 (1)	4 (< 1)
Headache	4 (< 1)	10 (2)	4 (1)	6 (2)	7 (2)	2 (< 1)
Sinusitis	2 (< 1)	4 (< 1)	5 (1)	6 (2)	3 (< 1)	4 (< 1)
Hypertension	6 (1)	4 (< 1)	3 (< 1)	2 (< 1)	8 (2)	2 (< 1)
Nausea	2 (< 1)	7 (2)	3 (< 1)	2 (< 1)	4 (1)	3 (< 1)
Worsening of COPD symptoms^b^	6 (1)	6 (1)	6 (2)	2 (< 1)	4 (1)	4 (< 1)

^a^AEs occurring in ≥2% of patients in any treatment group; ^b^that did not meet the definition of an exacerbation

*AE* adverse event, *COPD* chronic obstructive pulmonary disease, *SABA* short-acting β_2_-agonist, *SAE* serious adverse events, *SAL* salmeterol, *UMEC* umeclidinium, *URTI* upper respiratory tract infection, *VI* vilanterol

## Discussion

This analysis of the EMAX trial investigated whether baseline SABA use in patients with COPD is associated with the level of response to dual- versus mono-bronchodilation. Compared with UMEC/VI, a greater proportion of patients receiving UMEC or SAL withdrew from study treatment in the high SABA use subgroup. Both high and low SABA use subgroups demonstrated similar improvements in lung function with UMEC/VI versus UMEC. Patients with low baseline SABA use demonstrated significant incremental COPD symptom improvements, measured using SAC-TDI and E-RS scores, with UMEC/VI compared with UMEC. However, smaller and non-significant treatment differences were observed in the high SABA use subgroup with UMEC/VI versus UMEC for both symptom outcomes, despite a higher symptom burden in the high versus low SABA use subgroup at baseline. In contrast, the high SABA use subgroup showed significant reductions in daily rescue medication use that exceeded the non-significant reductions seen in the low SABA use subgroup; however, this may be in part due to patients in the low baseline SABA subgroup having little potential for improvement in this endpoint, since the majority were not using SABA at baseline. The findings with UMEC/VI versus UMEC might suggest that treatment differences may be impacted by concurrent use of rescue medication, with diminished efficacy of the LABA component observed on subjective symptom-based patient-reported outcomes in patients with high SABA use (approximately 4 puffs/day based on the fractional polynomial analyses) at baseline, whereas the effect was not so marked with UMEC/VI versus SAL (i.e. the addition of LAMA).

There are several possible mechanisms for the observed impact of SABA use on symptom outcomes. One possibility is the development of tolerance to β_2_-agonist bronchodilators or a loss of responsiveness to LABA maintenance bronchodilators [12]. However, as the improvements in change from baseline in lung function and symptoms were greater in high versus low SABA subgroups for all maintenance regimens, it is unlikely that tolerance to β_2_-agonist bronchodilators, or a loss of responsiveness to their effects, explain the diminished efficacy differences between LAMA/LABA and LAMA therapy seen in the current analyses. There could be pathophysiological differences between high and low SABA users that are unaccounted for in our analyses and that cannot be adjusted for or explained. It has previously been suggested that SABA use may be habitual [13]; however, our data do not support this, as differences in baseline disease characteristics suggest that the high SABA use subgroup had more severe COPD (worse lung function, a higher symptom burden and increased exacerbation incidence) compared with the low SABA use subgroup, and fewer were using long-acting maintenance therapy at baseline. Furthermore, in the high SABA use subgroup, the rates of study treatment withdrawal were higher for patients receiving monotherapy compared with UMEC/VI. This is in agreement with other studies that indicate that SABA use may be considered a marker of disease severity and increased exacerbation risk [1, 5, 7] or a marker of suboptimal care.

An intriguing hypothesis to consider is that high SABA use in addition to a LAMA therapy may blunt the patient-perceived symptom response to LAMA/LABA therapy. Patients using high levels of SABA at baseline and retaining high SABA use on LAMA monotherapy, may not be able to distinguish between the benefit of frequent SABA and daily LABA therapy, and therefore not perceive any additional change in symptom burden in response to dual therapy, despite an improvement in lung function and a reduction in SABA use. This is supported by the finding that the between treatment differences (UMEC/VI vs either monotherapy) in SAC-TDI and change from baseline in E-RS were smaller in the high SABA use subgroup compared with the low SABA use subgroup. As such, in patients with high SABA use, symptomatic treatment response may be more effectively monitored by assessing change in rescue medication use.

In the subgroup analyses, similar findings to those observed for UMEC/VI versus UMEC were also evident when comparing symptom outcomes between UMEC/VI and SAL, with symptom improvements generally more favourable for the dual therapy in the low baseline SABA use subgroup than in the high SABA use subgroup. However, UMEC/VI was found to provide significant improvements in E-RS score and exacerbation risk reduction versus SAL in both the high and low SABA use subgroups. This suggests that high SABA use may have less of an impact upon treatment differences between LAMA/LABA versus LABA than between LAMA/LABA and LAMA. Consequently, it is possible that when interpreting symptom outcomes between different classes of maintenance bronchodilators, the type of short-acting therapy, anticholinergic or β_2_−agonist bronchodilator therapy, and level of concurrent SABA use could increase the complexity of data interpretation.

The potential for confounding of LABA and SABA bronchodilator efficacy on perceived dyspnoea and exacerbation outcomes with increased baseline SABA use is consistent with a previous post hoc analysis by Naya et al of two large randomised controlled trials [1]. In the post hoc analysis, the levels of β_2_−agonist rescue medication use were higher than in the EMAX trial and almost half the population were using concurrent ICS, whereas ICS use was not permitted in the EMAX trial [1]; however, the findings presented here from a prospective analysis of the EMAX trial are generally supportive of the findings by Naya et al [1]. Together these data suggest the need for consideration of SABA use when assessing the incremental symptom benefits observed with dual- versus mono-bronchodilator therapy.

Assessing outcomes using fractional polynomial modelling allows modelling of a non-linear relationship. This provides a wider assessment of treatment differences between maintenance therapies across covariate values than may be observed compared with pre-determined subgroups. The use of fractional polynomial analysis in this study provides more informative results than the subgroup analysis alone and indicates that the need to consider SABA use is of particular importance to patients who use high levels of SABA (> 4 puffs/day). Similar approaches have recently been used to compare the efficacy of triple ICS/LAMA/LABA therapy versus LAMA/LABA according to baseline blood eosinophil count [14], and to evaluate the exacerbation risk reduction efficacy of budesonide–formoterol versus formoterol according to baseline blood eosinophil count [15]. Analysing SABA use with this modelling approach may be more beneficial for physicians to determine how to optimise treatment for their patients. This may be particularly important in maintenance-naïve patients with symptomatic COPD receiving short-acting bronchodilators alone, who may have developed a reliance on rescue bronchodilator therapy.

The following limitations should be considered when interpreting the results of these analyses. As there is currently no consensus for what constitutes low or high SABA use among patients with COPD, and level of use was unknown pre-study, we pre-specified the median SABA use (1.5 puffs/day) as the subgroup threshold to generate numerically balanced subgroups. The high and low SABA subgroups were nonetheless large and baseline clinical characteristics were well balanced between treatment arms within each subgroup. Also, as approximately a third of patients were not using SABA at baseline, an analysis comparing three subgroups of no, low and high SABA users may be of interest; however, this post hoc analysis would result in small, potentially less well-balanced subgroups and would provide little additional information than that already provided by the fractional polynomial analyses, which include estimates of efficacy differences for non-SABA users. The EMAX population had a low risk of exacerbation; as a consequence of this and the relatively short study duration, the subgroup analyses were not powered to detect exacerbation treatment differences, and so assessment of the impact of high versus low SABA use on the risk of exacerbation was limited. As VI is not available as a licensed drug, SAL was used as the comparator LABA in this study. However, evidence suggests that the efficacy of VI and SAL are similar on trough FEV_1_ and TDI [16]. Finally, the fractional polynomial analysis suggests that most of the diminished treatment differences for symptom-based outcomes between the LAMA/LABA and LAMA monotherapy were observed in the 18% of EMAX patients who used ≥4 puffs/day of SABA; consequently our findings should not be generalised to populations with less frequent SABA use.

## Conclusions

The results of this analysis have several potentially important implications for clinical practice and clinical trials. The findings indicate that it may be more difficult to demonstrate symptomatic treatment benefits between maintenance bronchodilator classes in patients with high SABA use. Therefore, SABA use should be considered as a potential confounding variable when designing and interpreting clinical trials comparing different classes of maintenance bronchodilators. Additional research is required to confirm this finding and the mechanism by which it occurs. The data also provide further evidence that high SABA use is a marker of more severe and symptomatic disease. As such, physicians should consider more intensive maintenance therapies for patients with high SABA use, particularly as several studies have demonstrated that high rescue medication use is associated with worse disease outcomes [5, 7].

## Supplementary information

**Additional file 1.** Distribution of baseline SABA use (puffs/day)

**Additional file 2.** Study treatment withdrawal

## Data Availability

Anonymised individual participant data and study documents can be requested for further research from www.clinicalstudydatarequest.com.

## Abbreviations

AE: Adverse event
BDI: Baseline Dyspnoea Index
CAT: COPD Assessment Test
CFB: Change from baseline
CI: Confidence interval
COPD: Chronic obstructive pulmonary disease
EMAX: Early MAXimisation of bronchodilation for improving COPD stability
E-RS: Evaluating Respiratory Symptoms
COPD; FEV_1_: Forced expiratory volume in 1 s
FP1: First-degree fractional polynomial
FP2: Second-degree fractional polynomial
GADS: Global assessment of disease severity
GOLD: Global Initiative for Chronic Obstructive Lung Disease
HR: Hazard ratio
ICS: Inhaled corticosteroids
ITT: Intent-to-treat
LABA: Long-acting β_2_-agonist
LAMA: Long-acting muscarinic antagonist
LS: Least squares
OR: Odds ratio
RR: Risk reduction
SD: Standard deviation
SABA: Short-acting β_2_-agonist
SAC-TDI: Self-administered computerised Transition Dyspnoea Index
SAE: Serious adverse event
SAL: Salmeterol
SGRQ: St George’s Respiratory Questionnaire
TDI: Transition Dyspnoea Index
UMEC: Umeclidinium
VI: Vilanterol

## References

[1] Naya I, Lipson DA, Boucot I, Gakava L, Compton C (2019). Impact of prior and concurrent medication on exacerbation risk with long-acting bronchodilators in chronic obstructive pulmonary disease: a post hoc analysis. Respir Res.

[2] Donohue JF, Jones PW, Bartels C, Marvel J, D'Andrea P, Banerji D (2018). Correlations between FEV1 and patient-reported outcomes: a pooled analysis of 23 clinical trials in patients with chronic obstructive pulmonary disease. Pulm Pharmacol Ther.

[3] Dransfield MT, Bailey W, Crater G, Emmett A, O'Dell DM, Yawn B (2011). Disease severity and symptoms among patients receiving monotherapy for COPD. Prim Care Respir J.

[4] European Medicines Agency (2012). Guideline on clinical investigation of medicinal products in the treatment of chronic obstructive pulmonary disease (COPD).

[5] Jenkins CR, Postma DS, Anzueto AR, Make BJ, Peterson S, Eriksson G (2015). Reliever salbutamol use as a measure of exacerbation risk in chronic obstructive pulmonary disease. BMC Pulmon Med.

[6] Punekar YS, Naya I, Small M, Holbrook T, Wood R, Mullerova H (2017). Bronchodilator reliever use and its association with the economic and humanistic burden of COPD: a propensity-matched study. J Med Econ.

[7] Punekar YS, Sharma S, Pahwa A, Takyar J, Naya I, Jones PW (2017). Rescue medication use as a patient-reported outcome in COPD: a systematic review and regression analysis. Respir Res.

[8] Maltais F, Bjermer L, Kerwin EM, Jones PW, Watkins ML, Tombs L (2019). Efficacy of umeclidinium/vilanterol versus umeclidinium and salmeterol monotherapies in symptomatic patients with COPD not receiving inhaled corticosteroids: the EMAX randomised trial. Respir Res.

[9] Celli BR, MacNee W (2004). Standards for the diagnosis and treatment of patients with COPD: a summary of the ATS/ERS position paper. Eur Respir J.

[10] Jones PW (2002). Interpreting thresholds for a clinically significant change in health status in asthma and COPD. Eur Respir J.

[11] Leidy NK, Murray LT, Monz BU, Nelsen L, Goldman M, Jones PW (2014). Measuring respiratory symptoms of COPD: performance of the EXACT- respiratory symptoms tool (E-RS) in three clinical trials. Respir Res.

[12] Cazzola M, Page CP, Calzetta L, Matera MG (2012). Pharmacology and therapeutics of bronchodilators. Pharmacol Rev.

[13] George M (2018). Adherence in asthma and COPD: new strategies for an old problem. Respir Care.

[14] Pascoe S, Barnes N, Brusselle G, Compton C, Criner GJ, Dransfield MT (2019). Blood eosinophils and treatment response with triple and dual combination therapy in chronic obstructive pulmonary disease: analysis of the IMPACT trial. Lancet Respir Med.

[15] Bafadhel M, Peterson S, De Blas MA, Calverley PM, Rennard SI, Richter K (2018). Predictors of exacerbation risk and response to budesonide in patients with chronic obstructive pulmonary disease: a post-hoc analysis of three randomised trials. Lancet Respir Med.

[16] Donohue JF, Betts KA, Du EX, Altman P, Goyal P, Keininger DL (2017). Comparative efficacy of long-acting β2-agonists as monotherapy for chronic obstructive pulmonary disease: a network meta-analysis. Int J Chron Obstruct Pulm Dis.

