# Supplementary figures and images for: Salbutamol use in relation to maintenance bronchodilator efficacy in COPD: a prospective subgroup analysis of the EMAX trial
Source: Respir Res. 2020 Oct 22;21:280. doi: 10.1186/s12931-020-01451-8 (PMC7579818; doi:10.1186/s12931-020-01451-8)

**Additional File 1**: Distribution of baseline SABA use (puffs/day)


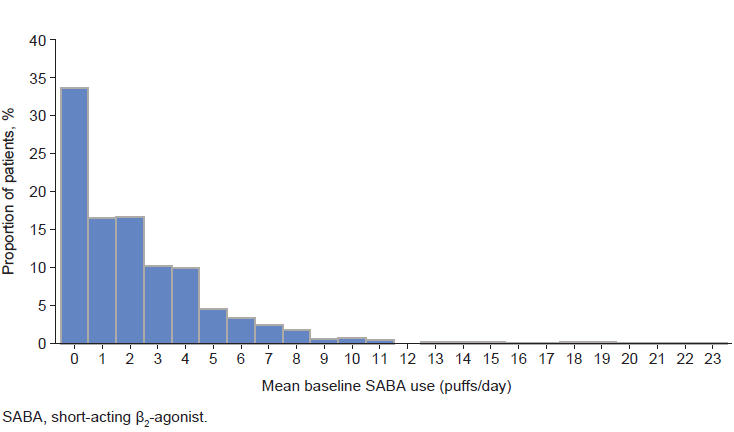

Supplement: Supplementary file 1 — Additional file 1. Distribution of baseline SABA use (puffs/day) [file 12931_2020_1451_MOESM1_ESM.docx]
