# Supplementary material for: Salbutamol use in relation to maintenance bronchodilator efficacy in COPD: a prospective subgroup analysis of the EMAX trial
Source: Respir Res. 2020 Oct 22;21:280. doi: 10.1186/s12931-020-01451-8 (PMC7579818; doi:10.1186/s12931-020-01451-8)
Supplement: Supplementary file 2 — Additional file 2. Study treatment withdrawal [file 12931_2020_1451_MOESM2_ESM.docx]

**Additional File 2: Study treatment withdrawal**

|  | **High baseline SABA (≥1.5 puffs/day)** | | | **Low baseline SABA (<1.5 puffs/day)** | | |
| --- | --- | --- | --- | --- | --- | --- |
|  | **UMEC/VI** | **UMEC** | **SAL** | **UMEC/VI** | **UMEC** | **SAL** |
| Study treatment withdrawal, n/N (%) | 50/415 (12) | 90/401 (22) | 71/396 (18) | 46/395 (12) | 60/399 (15) | 54/412 (13) |
| UMEC/VI vs comparator, hazard ratio (95% CI) | - | 0.51 (0.36, 0.72); p<0.001 | 0.66 (0.46, 0.95); p=0.024 | - | 0.77 (0.52, 1.13); p=0.174 | 0.85 (0.57, 1.25); p=0.406 |

CI, confidence interval; SABA, short-acting β_2_-agonist; SAL, salmeterol; UMEC, umeclidinium; VI, vilanterol.
